# Tradeoff-in-the-Nephron: A Theory to Explain the Primacy of Phosphate in the Pathogenesis of Secondary Hyperparathyroidism

**DOI:** 10.3390/nu9050427

**Published:** 2017-04-26

**Authors:** Kenneth R. Phelps

**Affiliations:** 1 Research Service, Stratton Veterans’ Affairs Medical Center, Albany, NY 12208, USA; kenneth.phelps@va.gov; Tel.: +1-518-626-5641 or +1-518-626-5622; Fax: +1-518-626-5628; 2 Department of Medicine, Division of Nephrology, Albany Medical College, Albany, NY 12208, USA

**Keywords:** chronic kidney disease, secondary hyperparathyroidism, phosphate, calcium, parathyroid hormone, cortical distal nephron, distal convoluted tubule

## Abstract

Chronic kidney disease (CKD) causes secondary hyperparathyroidism (SHPT). The cardinal features of SHPT are persistence of normocalcemia as CKD progresses and dependence of the parathyroid hormone concentration ([PTH]) on phosphate influx (I_P_). The tradeoff-in-the-nephron hypothesis integrates these features. It states that as the glomerular filtration rate (GFR) falls, the phosphate concentration ([P]_CDN_) rises in the cortical distal nephron, the calcium concentration ([Ca]_CDN_) in that segment falls, and [PTH] rises to maintain normal calcium reabsorption per volume of filtrate (TR_Ca_/GFR). In a clinical study, we set GFR equal to creatinine clearance (C_cr_) and I_P_ equal to the urinary excretion rate of phosphorus (E_P_). We employed E_P_/C_cr_ as a surrogate for [P]_CDN_. We showed that TR_Ca_/C_cr_ was high in patients with primary hyperparathyroidism (PHPT) and normal in those with SHPT despite comparably increased [PTH] in each group. In subjects with SHPT, we examined regressions of [PTH] on E_P_/C_cr_ before and after treatment with sevelamer carbonate or a placebo. All regressions were significant, and ∆[PTH] correlated with ∆E_P_/C_cr_ in each treatment cohort. We concluded that [P]_CDN_ determines [PTH] in CKD. This inference explains the cardinal features of SHPT, much of the evidence on which other pathogenic theories are based, and many ancillary observations.

## 1. Introduction

Chronic kidney disease (CKD) causes the parathyroid hormone concentration ([PTH]) to rise to abnormally high values. This phenomenon, secondary hyperparathyroidism (SHPT), begins early in the course of CKD and increases in prevalence and severity as the glomerular filtration rate (GFR) falls [1,2,3,4,5]. A secondary skeletal lesion, osteitis fibrosa, evolves with SHPT and presumably contributes to the increased fracture risk of patients with CKD [6,7]. Excessive PTH may also play a role in extraskeletal manifestations of uremia [8,9]. 

SHPT exhibits two reproducible characteristics: the ionized calcium concentration ([Ca]_i_) is consistently physiologic until GFR is severely reduced [1,3], and [PTH] varies directly and substantially with phosphate influx (I_P_). In experimental CKD, [PTH] is elevated at customary I_P_ but falls to normal if I_P_ is reduced in proportion to GFR [10,11,12,13,14]. We have not found a reported exception to this rule.

The pathogenesis of SHPT is unresolved. In this paper we present a hypothesis, tradeoff-in-the-nephron, that integrates the primacy of I_P_ with the paradox of normal [Ca]_i_ and high [PTH]. The hypothesis is compatible with evidence on which other pathogenic theories are based, and it illuminates many ancillary observations. We suggest that resistance to the calcemic action of PTH arises in the cortical distal nephron (CDN), where PTH regulates calcium reabsorption [15]. An increased phosphate concentration at that site ([P]_CDN_) reduces the concentration of calcium ([Ca]_CDN_) through formation of complexes, and secondarily necessitates high [PTH] to maintain normal [Ca]_i_ [16,17,18]. Since tradeoff-in-the-nephron depends entirely on inferred events in glomerular filtrate, we emphasize that the hypothesis pertains only to CKD that does not require dialysis. Abbreviations are defined at the end of the paper.

## 2. Explications of Secondary Hyperparathyroidism: A Chronology

### 2.1. The Primacy of Phosphate Influx

We define influx of phosphate (I_P_) as the net rate of phosphate flow from all sources into extracellular fluid. When plasma is in equilibrium with respect to phosphate, I_P_ determines, equals, and is measurable as the urinary excretion rate, E_P_ [19,20,21]. At any GFR, in animals or humans, [PTH] varies promptly and directly with oral or intravenous I_P_ [22,23,24,25,26,27,28,29,30,31,32,33,34,35,36,37]. If a change in I_P_ persists, the resulting change in [PTH] also persists [23,24,26,31,34,36,37].

[Ca]_i_ or the total serum calcium concentration ([Ca]_s_) may vary inversely with I_P_ [22,24,32], but I_P_ affects [PTH] whether calcemia changes perceptibly or not [12,13,14,28,30,33,34,35,36,37]. The serum phosphorus concentration ([P]_s_) may vary directly with I_P_ [17,18], but I_P_ affects [PTH] whether [P]_s_ changes or not [36,37]. SHPT is often associated with glandular hyperplasia, but reduction of I_P_ normalizes [PTH] despite persistence of hyperplasia [28,29]. When the loss of GFR is modest, high [PTH] may coincide with low-normal [P]_s_ at normal E_P_ [36,38], and an oral bolus of phosphate may raise [PTH] even though [P]_s_ falls [32]. In disorders characterized by impaired proximal tubular phosphate reabsorption, high I_P_ induces SHPT even if low [P]_s_ persists [39]. 

In the 1970s, Slatopolsky and colleagues reported that extreme limitation of I_P_ prevented SHPT in 5/6 nephrectomized dogs, and subsequently showed that reduction of dietary phosphate in proportion to GFR produced an identical result [10,11]. In the same model, Kaplan and colleagues documented reversal of established SHPT with proportional phosphate restriction [12]; subsequently, other investigators duplicated or approximated this result in animals and humans [13,14,24,33,34,35,36,37,40]. Although a reduction in I_P_ increased the concentration of 1,25-dihydroxyvitamin D (1,25D) in mild CKD [35,36], the same intervention lowered [PTH] without raising [1,25D] in more advanced disease [13,14,33,34,40,41].

### 2.2. The Original Tradeoff Hypothesis

Bricker proposed the following sequence of events to explain the role of phosphate in SHPT [42]: intake and gastrointestinal absorption of phosphate continue unabated as nephrons are lost; a temporary rise in plasma phosphate ([P]_p_) reduces [Ca]_i_ through formation of complexes; parathyroid cells sense this reduction and raise [PTH] in response; increased [PTH] restores normal [Ca]_i_ through actions on target organs and simultaneously corrects [P]_p_ by reducing tubular phosphate reabsorption. A “tradeoff” thus occurs in which SHPT is the price paid for normal [Ca]_i_ and [P]_p_. 

Eventually, evidence appeared that was discordant with Bricker’s synthesis. Investigators identified patients with what would now be called Stage 3 CKD in whom [PTH] was increased despite low-normal [P]_s_ [36,38], and oral phosphate raised [PTH] in such patients even though [P]_s_ fell simultaneously [32]. In patients with hypophosphatemia due to impaired phosphate reabsorption, high I_P_ raised [PTH] without correcting [P]_s_ [39]. In vitro, modest increments in [P]_p_ did not reduce [Ca]_i_ [43]. 

### 2.3. Skeletal Resistance to PTH

As Slatopolsky, Kaplan, and their colleagues were linking SHPT to I_P_, others focused on the paradox of high [PTH] and normal [Ca]_i_. A source of calcium seemed resistant to PTH, and the skeleton was assumed to be that source. We have found no evidence that the CDN was considered. 

Massry and colleagues measured effects of infused parathyroid extract (PTE) on the serum calcium concentration ([Ca]_s_) in humans. PTE raised [Ca]_s_ by more than 1.0 mg/dL in subjects with normal GFR and by approximately 0.5 mg/dL in patients with mild, advanced, or end-stage renal disease [44]. Llach and colleagues examined responses to endogenous PTH by infusing the chelating agent ethylenediaminetetraacetic acid (EDTA); in comparison to control subjects, patients with mild CKD responded to EDTA with more severe hypocalcemia, much higher [PTH], and a more delayed recovery of [Ca]_s_ [45]. 

Three hypotheses were offered to explain the blunted calcemic response in CKD: a deficiency of 1,25-dihydroxyvitamin D (1,25D) undermined the effect of PTH on osteolysis; circulating phosphate mediated skeletal resistance by an unknown mechanism; and chronically increased [PTH] down-regulated PTH receptors in bone. In dogs made uremic by ureteral ligation or nephrectomy, preliminary administration of 1,25D improved but did not normalize the calcemic response to PTE [46]. Somerville and Kaye found that 1,25D ameliorated PTH resistance in chronic but not acute renal failure [47]; in contrast, phosphate was the agent of resistance when uremia was created by intravenous infusion of urine from intact kidneys [48]. In an isolated rat-tail preparation, the same investigators demonstrated that phosphate could inhibit calcium release from bone [49]. 

In 5/6 nephrectomized dogs, Kaplan and colleagues observed that neither 1,25D nor phosphate restriction could normalize the calcemic response to PTH even though each intervention restored it partially [50]. Rodriguez and colleagues also achieved partial improvement with these interventions but found that parathyroidectomy restored the calcemic response completely [41,51]. It should be noted that parathyroidectomized animals were maintained with a high-calcium diet post-operatively and a low-phosphate diet during the PTH infusion [51].

We are reluctant to attribute SHPT to skeletal resistance to PTH. If kidneys are functional, and if GFR is assumed to equal creatinine clearance (C_cr_), then the flux of calcium into plasma (I_Ca_) equals the urinary excretion rate (E_Ca_), and the impact of I_Ca_ on [Ca]_i_ is measurable as calcium excreted per volume of filtrate (E_Ca_/C_cr_) [16]. If the skeletal resistance theory is correct, we should not see normal [PTH] when E_Ca_/C_cr_ is low, or high [PTH] when E_Ca_/C_cr_ is high. However, we found normal [PTH] despite minimal E_Ca_/C_cr_ in some control subjects, and high [PTH] despite robust E_Ca_/C_cr_ in some patients with CKD [16]. Low I_Ca_ did not provoke SHPT at normal GFR, and high I_Ca_ did not prevent it at reduced GFR. We doubt that the skeleton is the principal site of PTH resistance in SHPT.

### 2.4. Deficiency of 1,25-Dihyroxyvitamin D

The active metabolite of vitamin D, 1,25-dihydroxyvitamin D (1,25D), is synthesized throughout the nephron [52]. Its concentration falls as nephrons are lost, and SHPT is widely attributed to this phenomenon [1,2,4,5]. In theory, a reduction in [1,25D] could necessitate a rise in [PTH] by compromising intestinal absorption and tubular reabsorption of calcium [15,35,53], but the preferred explanation for SHPT at present is loss of the suppressive effect of 1,25D on PTH gene transcription [54,55]. This attribute of the metabolite is the basis for treatment of SHPT with vitamin D receptor activators (VDRAs) [56]. 

Despite the calcemic and genomic effects of 1,25D, evidence from multiple sources suggests that low [1,25D] does not cause high [PTH] in CKD. Levin and colleagues found normal [PTH] and low [1,25D] in 13% of a large sample with CKD [4]. Some investigators found inverse relationships between [PTH] and [1,25D] [1,2,4,5], but we did not [16,17]. Although 1,25D appeared to mediate the interaction between I_P_ and [PTH] in mild CKD [35,36], high I_P_ increased [PTH] in an animal model when [1,25D] did not fall [57], and low I_P_ reduced [PTH] in advanced disease when [1,25D] did not rise [13,14,33,34,40,41]. If phosphate restriction can normalize [PTH] while [1,25D] remains suppressed, then deficiency of 1,25D cannot be the proximal cause of SHPT. 

### 2.5. Direct Stimulation of PTH Secretion by Circulating Phosphate

In 1996, two groups showed that parathyroid tissue from normal rats secreted PTH in proportion to the phosphate concentration ([P]) in culture medium [57,58]. Two years later, the observation was repeated with hyperplastic tissue from patients with SHPT [59]. Whereas changes in [Ca]_i_ altered [PTH] within one hour [58], changes in [P] did so over 3–5 h [57,58]. [P] did not affect PTH gene transcription [57,59]; observations by Moallem and colleagues suggested indirectly that cytosolic proteins stabilized PTH mRNA in response to high [P] [60]. 

Evidence of a direct relationship between [P]_s_ and [PTH] was also found in vivo. Takahashi, Slatopolsky, and their colleagues demonstrated strong linear correlations between [PTH] and [P]_s_ in rodents subjected to 5/6 nephrectomy [28,57]. Kates and colleagues confirmed a similar relationship in humans with CKD, but it was demonstrable only in subjects with serum creatinine ([cr]_s_) ≤3.0 mg/dL [61]. On some occasions our group also found significant linear regressions of [PTH] on [P]_s_ [18].

We do not doubt that hyperphosphatemia increases PTH synthesis in CKD. However, when kidneys are functional, correlations between [P]_s_ and [PTH] may reflect dependence of both concentrations on a third variable. If E_P_ and TR_P_ are rates of excretion and tubular reabsorption of phosphorus, [P]_s_ equals the sum of E_P_/C_cr_ and TR_P_/C_cr_ [19]. E_P_/C_cr_ quantifies the contribution of I_P_ to [P]_s_, but it also serves as a mathematical surrogate for [P]_CDN_, which we believe to be the principal determinant of [PTH] in CKD [17,18]. In patients with Stage 3 and 4 CKD, we found that [PTH] varied directly with E_P_/C_cr_ and [P]_s_ before administration of sevelamer or a placebo, but with E_P_/C_cr_ alone after treatment [17]. We therefore attributed the correlation of [PTH] with [P]_s_ to a dependence of both concentrations on E_P_/C_cr_ [18]. In our study and that of Kates and colleagues, most values of [P]_s_ were in the normal range and were in fact lower than many fasting values of control subjects without SHPT [17,18,61,62]. Consequently, we suspect that [P]_CDN_, as represented by E_P_/C_cr_, determined [PTH] in both studies. When kidneys are functional, the putative effect of [P]_s_ on [PTH] cannot be separated from that of [P]_CDN_. 

### 2.6. Impaired Suppression of the PTH Gene by Fibroblast Growth Factor 23 (FGF23)

FGF23 is a hormone made predominantly but not exclusively by osteocytes [63,64]. In CKD, its concentration is already increased when [PTH] begins to rise [32,65]. Its effects on parathyroid glands and renal tubules are initiated by simultaneous binding to a cognate receptor, FGFR1c, and a co-receptor, the membrane form of klotho [66]. When GFR is normal, FGF23 suppresses transcription of the PTH gene [67], but this action dissipates as GFR falls because FGFR1c and klotho recede in parathyroid tissue [68,69]. 

PTH and FGF23 reduce proximal tubular phosphate reabsorption by promoting removal of sodium-phosphate co-transporters from the brush border membrane [66], and both hormones increase calcium reabsorption in the distal convoluted tubule [70]. The actions of the two hormones are thought to be integrated at both sites, and both may be required to maintain normal [P]_s_ and [Ca]_i_ in CKD [70,71,72]. In theory, it is possible that the loss of the genomic effect of FGF23 in parathyroid tissue facilitates synthesis of PTH in CKD. It is also possible that the calcium-reabsorbing action of FGF23 promotes reversal of SHPT when I_P_ is reduced in proportion to GFR [12,13,14,29].

### 2.7. Deficiency of 25-Hydroxyvitamin D (25D)

Although definitions of vitamin D insufficiency and deficiency are debated, [25D] ≥30 ng/mL (74.9 nmol/L) is generally accepted as evidence of full repletion [73,74,75,76]. Nevertheless, in CKD, use of vitamin D supplements to achieve [25D] of 30–40 ng/mL (99.8 nmol/L) has yielded marginal reductions of [PTH] [77,78,79]. To examine effects of higher [25D], Sprague and colleagues administered three doses of extended-release calcifediol [25D] to subjects with CKD [80]. A dose of 30 mcg/day achieved a mean [25D] of 37.3 ng/mL (93.1 nmol/L) and a 20.9% reduction in [PTH]; corresponding results of 60 and 90 mcg/day were [25D] of 66.9 and 84.8 ng/mL (167.0 and 211.7 nmol/L) and reductions in [PTH] of 32.8% and 39.3%, respectively. [1,25D] rose with the dose of 25D. The effect of [25D] between 30 and 40 ng/mL was again modest, and the response to higher doses was incomplete. A more protracted trial yielded qualitatively similar results [81]. Although ample doses of 25D induce partial reversal of SHPT, vitamin D insufficiency is not the primary cause of SHPT in CKD.

## 3. Tradeoff-in-the-Nephron

The ultrafilterable fraction of plasma calcium (Ca_uf_) consists of Ca_i_ and a small amount bound to organic anions in complexes [82]. In normal health, [Ca]_uf_ is maintained by influx from the gastrointestinal tract and by tubular reabsorption of filtered calcium. I_Ca_ determines and equals E_Ca_ [16]. 

The filtration rate of calcium, (GFR)[Ca]_uf_, is the sum of its excretion and reabsorption rates:

(1) GFR[Ca]_uf_ = E_Ca_ + TR_Ca_. Division by GFR yields a formula for [Ca]_uf_:
(2) [Ca]_uf_ = E_Ca_/GFR + TR_Ca_/GFR. If creatinine clearance (C_cr_) is assumed to equal GFR, then:
(3) [Ca]_uf_ = E_Ca_/C_cr_ + TR_Ca_/C_cr_ = [Ca]_u_[cr]_s_/[cr]_u_ + TR_Ca_/C_cr_. It follows that:
(4) TR_Ca_/C_cr_ = [Ca]_uf_ − E_Ca_/C_cr_ = [Ca]_uf_ − [Ca]_u_[cr]_s_/[cr]_u_ [16].

At both normal and reduced GFR, [Ca]_uf_ is on average 0.4–0.6 mg/dL greater than [Ca]_i_ [16,82]. In our experience, mean [Ca]_i_ of 5.0 mg/dL (1.25 mmol/L) was accompanied by mean [Ca]_uf_ of 5.4 mg/dL. Since I_Ca_ and E_Ca_ fell in tandem with GFR, E_Ca_/C_cr_ and TR_Ca_/C_cr_ approximated 0.1 mg/dL and 5.3 mg/dL at any GFR [16].

**Figure 1:**
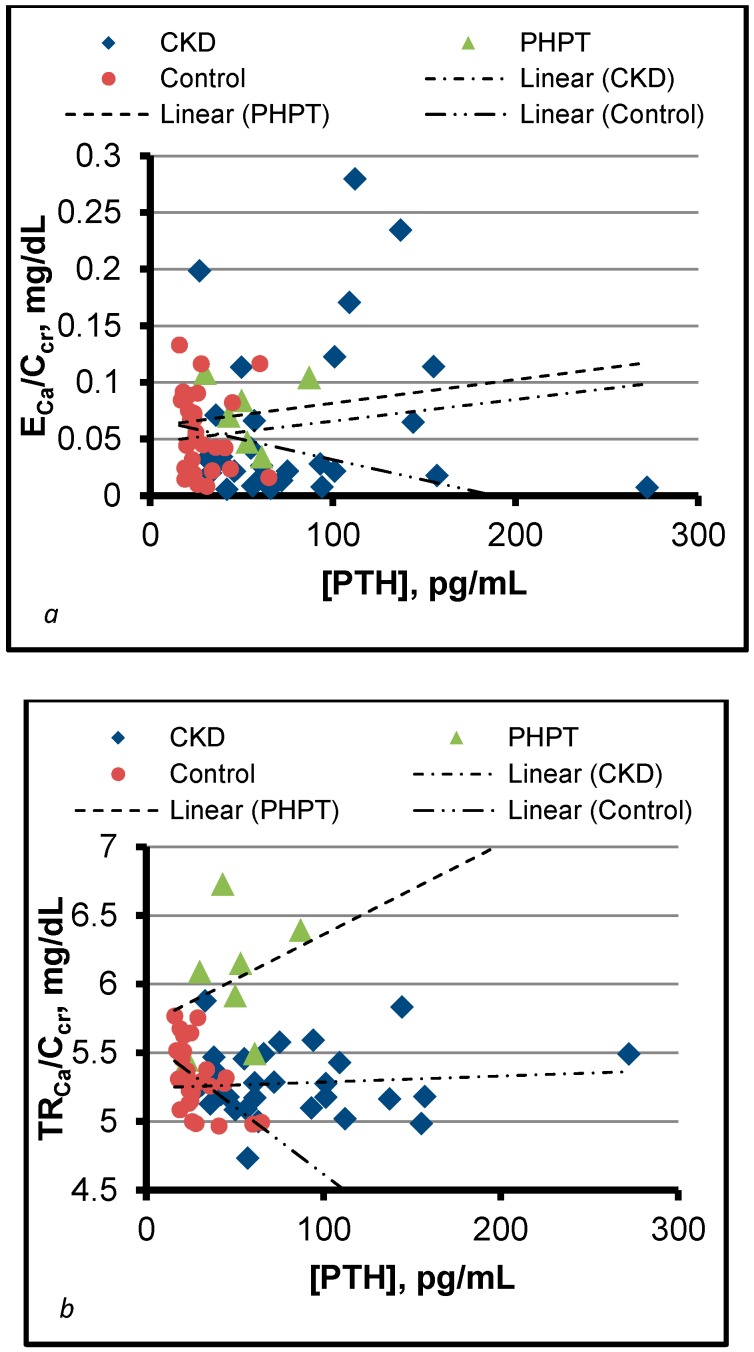
Plots of E_Ca_/C_cr_ and TR_Ca_/C_cr_ against [PTH] in control subjects and patients with primary and secondary hyperparathyroidism (PHPT and SHPT). All data are derived from morning fasting specimens of urine and serum or plasma. Circles represent normal controls. Triangles and diamonds represent patients with PHPT and SHPT (CKD), respectively. Frame (**a**) shows that the lowest recorded values of E_Ca_/C_cr_ in controls were compatible with normal [PTH]. It also shows that a minority of patients with CKD exhibited high E_Ca_/C_cr_ and high [PTH] simultaneously. Frame (**b**) shows that [PTH] capable of causing high TR_Ca_/C_cr_ in patients with PHPT maintained normal TR_Ca_/C_cr_ in patients with CKD. Reproduced from [16] with permission of the publisher (Dustri-Verlag). E_Ca_, Urinary excretion rate of calcium, mass/time; C_cr_, Creatinine clearance (volume/time); TR_Ca_, Rate of tubular reabsorption of calcium, mass/time; PTH, Parathyroid hormone; CKD, Chronic kidney disease.

We used Equation (4) to examine TR_Ca_/C_cr_ as a function of [PTH] in seven patients with primary hyperparathyroidism (PHPT), 29 patients with CKD (mean MDRD estimated GFR of 29.5 mL/min/1.73 m^2^, range 14–49), and 28 controls with normocalcemia and estimated GFR >60 mL/min/1.73 m^2^ [16]. Because of wide dispersion around mean values, [PTH] was not significantly different in PHPT and SHPT even though the 11 highest values in the study were seen in the latter, but concentrations were significantly higher in both of these groups than in controls. Fasting E_Ca_/C_cr_, the measurable consequence of calcium influx, was comparable in all three groups. This finding led to the conclusion that increased TR_Ca_/C_cr_, not increased I_Ca_, had caused hypercalcemia in PHPT [16]. Simultaneously, the results showed that [PTH] sufficient to increase TR_Ca_/C_cr_ in PHPT had maintained normal TR_Ca_/C_cr_ in SHPT (Figure 1). We therefore inferred that the CDN is partially resistant to the calcemic effect of PTH in CKD [16].

We reasoned that under conditions of reduced GFR and normal I_P_ (measurable as E_P_), the concentration of phosphate in the CDN ([P]_CDN_) would be greater than normal, as Bank and colleagues had demonstrated by micropuncture [83]. We hypothesized that high [P]_CDN_ would reduce the availability of Ca for reabsorption through the formation of soluble complexes or crystals, and would, thereby, necessitate increased [PTH] to maintain normal TR_Ca_/C_cr_, [Ca]_uf_, and [Ca]_i_. We believed that this hypothesis would elucidate the role of phosphate influx in the pathogenesis of SHPT and would explain the persistence of normocalcemia despite high [PTH] in CKD. 

Supporting evidence for the hypothesis was available. Tiselius and colleagues had argued that distal tubular filtrate is normally supersaturated with calcium-phosphate compounds, and had shown with in vitro simulations that calcium-phosphate crystals would be the first to form in the CDN after addition of calcium [84,85]. In rats subjected to ¾ nephrectomy, Haut and colleagues had found that a high-phosphate diet promoted calcium deposition in lumens and cells of cortical nephrons, and had shown that kidney calcium content rose on this diet even if [P]_s_ remained normal [86]. Biopsies had also revealed calcium deposition within CDNs of patients with phosphate-induced acute kidney injury [87]. Most importantly, treatment of SHPT with the calcimimetic agent cinacalcet had reduced [PTH], [Ca]_s_, and calcium reabsorption, but had not reduced E_Ca_ (or by inference, I_Ca_) [71].

We published evidence for the tradeoff-in-the-nephron hypothesis in 2014. Our underlying assumptions were that glomerular filtration of phosphate is virtually complete [88]; I_P_ determines and equals E_P_ at any GFR [19,20,21,25,35]; [P]_CDN_ rises at customary I_P_ as GFR falls [83]; and increased [P]_CDN_ promotes complexation of Ca as described above [84,85,86,87]. For simplicity, we also assumed that delivery of filtered phosphate to the CDN equals E_P_ even though phosphate may be secreted into the distal nephron in CKD [83,89].

Twenty-nine patients with eGFR of 14–49 mL/min/1.73 m^2^ participated in a study designed to examine the tradeoff-in-the-nephron hypothesis [17]. They were seen in a research clinic on five occasions, each separated by four weeks. Informed consent was obtained at the first visit, and patients who were taking intestinal phosphate-binding agents discontinued them at that time. A course of cholecalciferol was prescribed at the second visit to minimize any possible contribution of vitamin D deficiency to SHPT. Patients were instructed in a phosphate-restricted diet at the third visit and were asked to continue the diet through the end of the study. At the fourth visit, subjects were randomly assigned to a course of sevelamer carbonate or placebo with meals. Metabolic studies obtained at this visit revealed that the dietary instruction had been ineffective. Results of the therapeutic trial were ascertained at the fifth visit. 

We argued algebraically that E_P_/C_cr_ is proportional to [P]_CDN_ and hypothesized that [PTH] would therefore vary directly with E_P_/C_cr_ [17]. The purpose of sevelamer carbonate administration was to reduce this ratio. ∆E_P_/C_cr_ was negative in all sevelamer recipients, and the mean change was −0.5 ± 0.1 mg/dL. In placebo recipients, ∆E_P_/C_cr_ was evenly distributed over a range of positive and negative values, and the mean change was 0.04 ± 0.12 mg/dL. We interpreted dispersion around this mean as evidence of random variation in phosphate intake. 

**Figure 2:**
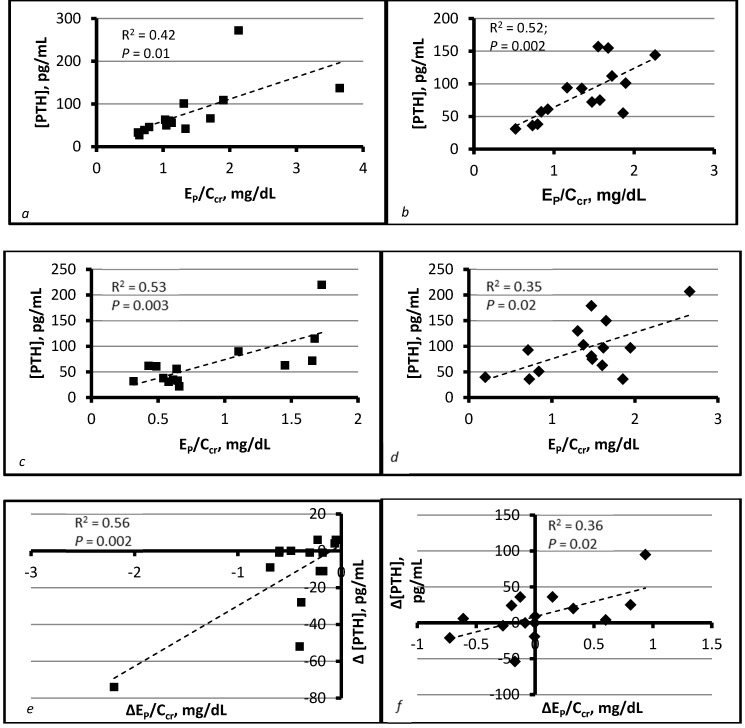
Relationship of [PTH] to E_P_/C_cr_ in sevelamer and placebo recipients. Squares pertain to the sevelamer group and diamonds to the placebo group. Graphs (**a**) and (**c**) show regressions of [PTH] on E_P_/C_cr_ before and after administration of sevelamer carbonate for four weeks. Graphs (**b**) and (**d**) show the same regressions before and after administration of a placebo for four weeks. Graphs (**e**) and (**f**) show regressions of ∆[PTH] on ∆E_P_/C_cr_ in the sevelamer and placebo groups, respectively, where “∆” = change during treatment. All regressions are statistically significant. Adapted from [17] with permission of the publisher (Dustri-Verlag). E_P_, urinary excretion rate of phosphorus, mass/time; C_cr_, creatinine clearance, volume/time.

In both groups, we found significant linear regressions of [PTH] on E_P_/C_cr_ and of ∆[PTH] on ∆E_P_/C_cr_ after treatment (Figure 2). Sevelamer recipients in whom ∆[PTH] did not vary with ∆E_P_/C_cr_ tended to have extremely low E_Ca_/C_cr_. The results supported the hypothesis that high [P]_CDN_ necessitates high [PTH] to achieve normal TR_Ca_/C_cr_, and also suggested that sufficient [Ca]_CDN_ is essential to the salutary effect of reduced I_P_ on [PTH] [17].

## 4. Compatibility of Tradeoff-in-the-Nephron with Existing Data

Tradeoff-in-the-nephron is a straightforward hypothesis. It states that high [P]_CDN_ reduces [Ca]_CDN_ by complexation and thus necessitates high [PTH] to maintain normal calcium reabsorption. [P]_CDN_ may rise as a consequence of high I_P_ at a normal GFR or normal I_P_ at a reduced GFR. The hypothesis implies that [PTH] rises in either circumstance, and this implication has been confirmed repeatedly [10,11,12,13,14,22,23,24,25,26,27,28,29,30,31,32,33,34,35,36,37,40,41,50]. Tradeoff-in-the-nephron explains the tight relationship of [PTH] to I_P_ in CKD and accounts for the requirement of high [PTH] to maintain normal TR_Ca_/C_cr_ and [Ca]_i_. If E_P_/C_cr_ is proportional to [P]_CDN_, it follows that [PTH] should be a recognizable function of E_P_/C_cr_. Our work has supported this inference [17,18].

In theory, calcium, 1,25D, or phosphate could affect the synthesis and release of PTH in CKD. Of these, only calcium regulates immediate secretion of stored hormone through its interaction with the membrane calcium receptor [90]. If I_P_ affects [PTH] by determining calcium availability for reabsorption, then changes in I_P_ should alter [PTH] quickly. In vivo and in vitro studies have confirmed this expectation [24,30,31,58].

Tradeoff-in-the-nephron explains why [PTH] was high as long as E_P_/C_cr_ was high [23,26] and low as long as E_P_/C_cr_ was low [34]. The hypothesis explains why [PTH] fell with I_P_ while hyperphosphatemia persisted [27,40]. It accounts for the chronicity of SHPT in CKD, in which [P]_CDN_ is continuously increased at normal I_P_ [83]. The hypothesis explains why [PTH] correlated with E_P_ but not [P]_s_ in early CKD [91], and with E_P_/C_cr_ but not [P]_s_ after administration of sevelamer or placebo [17]. It accounts for increased calciuria despite high [PTH] after an oral bolus of phosphate [32]. It explains why [PTH] was elevated in patients with mild CKD, normal I_P_ and low-normal [P]_s_ [35,36], and why [PTH] rose after a bolus of phosphate even though [P]_s_ fell simultaneously [32]. The hypothesis provides a mechanism for high [PTH] in response to high I_P_ despite persistent hypophosphatemia [39]. Most importantly, it predicts normalization of [P]_CDN_, E_P_/C_cr_, and [PTH] when I_P_ is reduced in proportion to GFR [10,11,12,13,14,17,24,28,29,30,50].

The principal alternatives to tradeoff-in-the-nephron involve skeletal resistance to the calcemic action of PTH, the effect of 1,25D to suppress transcription of the PTH gene, and direct stimulation of PTH synthesis and secretion by circulating phosphate. Much of the evidence for these theories is compatible with our hypothesis. In subjects with functioning kidneys, 1,25D could have enhanced the calcemic response to PTH through its independent effect on calcium reabsorption in the CDN [15]. In addition to limiting calcium egress from bone [41,49], phosphate could have introduced resistance to PTH in the CDN by the mechanism implied in our hypothesis. Instead of making bone more sensitive to PTH, parathyroidectomy could have necessitated a diet that ensured maximal calcium reabsorption from the CDN in response to the hormone [51].

Recurrent themes emerge from studies of the calcemic response to PTH. Typically, the magnitude of the response was less at reduced than at normal GFR, and preparatory phosphate restriction or 1,25D administration mitigated but did not eliminate this difference [38,41,44,45,46,47,50,51]. A notable exception occurred when I_P_ was brought to zero in a model of uremia that left kidneys intact; in that instance, the calcemic response was restored completely [48]. These observations make sense if PTH acted on the CDN as well as the skeleton to raise [Ca]_s_. When filtrate contained no phosphate, a full complement of nephrons permitted a normal response to PTH even though experimental animals were uremic [48]. In other studies, a deficit of nephrons imposed a limit on the response to PTH that neither phosphate restriction nor 1,25D could overcome [38,41,44,45,46,47,50,51]. 

The premise that the CDN is the site of PTH resistance is also supported by effects of the calcimimetic agent cinacalcet. In patients with Stage 3 and 4 CKD, the drug reduced [PTH] by 43.1%, but simultaneously kept mean [Ca]_s_ between 8.5 and 9.0 mg/dL even though E_Ca_ rose or remained unchanged [71]. Since I_Ca_ determined E_Ca_, and since I_Ca_ and TR_Ca_ maintain [Ca]_uf_ at a given GFR [16], it follows that reduction of [PTH] with cinacalcet led to reduction of TR_Ca_/GFR. High [PTH] was apparently required for reabsorption sufficient to maintain normocalcemia [71]. 

The capacity of VDRAs to suppress PTH gene transcription can be exploited before ESRD is reached [77,92], but efficacy of the intervention does not confirm reversal of pathogenesis. If deficiency of 1,25D were the cause of SHPT, then normal [PTH] would be incompatible with low [1,25D] in CKD. Numerous investigators have documented this combination after sufficient reduction of I_P_ [13,14,33,34,40,41], and tradeoff-in-the-nephron explains why the combination is possible.

E_P_/C_cr_ is a determinant of [P]_s_, and [P]_s_ is a linear function of E_P_/C_cr_ in CKD [18,20,28,57,61]. At the same time, E_P_/C_cr_ is approximately proportional to [P]_CDN_ [17,18]. If [PTH] varies directly with [P]_s_ in vivo, the reason may be that [PTH] also varies directly with E_P_/C_cr_. We suggest that this confounding association is responsible for correlations between [PTH] and [P]_s_ in Stage 3 and 4 CKD [18,61]. 

## 5. Therapeutic Implications of Tradeoff-in-the-Nephron

Tradeoff-in-the-nephron implies that [PTH] is normal if [P]_CDN_ is normal. E_P_/C_cr_ is our surrogate for [P]_CDN_. Since I_P_ determines E_P_, a reduction of I_P_ in proportion to GFR yields normal E_P_/C_cr_. Proportional reduction of I_P_ was precisely the intervention that prevented and reversed SHPT in animal models of CKD [10,11,12,13,14,50]. It follows that normalization of E_P_/C_cr_ should do the same for patients with SHPT. 

In the 1980s and 1990s, European investigators employed severe dietary phosphate restriction to reduce [PTH] in patients with CKD [33,34,40]. Today, in the United States, a similar result requires a drastic revision of eating habits, including avoidance of phosphate preservatives [93,94]. This effort is necessary because to date, the most successful human studies of intestinal phosphate binders have reduced E_P_ by 25%–50% and [PTH] by 13%–35% [37,95,96,97,98]. Our theory and many animal studies suggest that E_P_/C_cr_ must be reduced to normal to reverse SHPT completely; if GFR has been reduced by 80%, E_P_ must be reduced by 80%. In addition to diet and binders, blockade of sodium-hydrogen exchanger 3 (NHE3) and inhibition of the intestinal sodium-phosphate 2b co-transporter may ultimately be required to lower I_P_ sufficiently [99,100]. Our experience suggests that normal E_Ca_/C_cr_ must also be established [17,91]. Attainment of [25D] >30 ng/mL may reduce [PTH] modestly, but we endorse it for other reasons [74]. We presume that normalization of [PTH] is desirable, but concede that the point is debatable [101].

## 6. Conclusions

Discordant empiric observations undermine each of the major theories concerning the pathogenesis of SHPT. We have sought a unifying explanation for the two most consistent features of the syndrome, which are dependence of [PTH] on I_P_ and persistence of normal [Ca]_i_ until CKD is far advanced. Tradeoff-in-the-nephron accounts for these features. The hypothesis also provides alternate explanations for much of the evidence on which other theories are based, and it sheds light on numerous ancillary observations. It traces SHPT to high [P]_CDN_ and predicts normal [PTH] at normal E_P_/C_cr_. An abundance of evidence is consistent with this prediction. The veracity of tradeoff-in-the-nephron is testable in patients by rigorous but feasible interventions.

## Abbreviations

CKD	Chronic kidney disease
GFR	Glomerular filtration rate (volume/time)
C_cr_	Creatinine clearance (volume/time)
PTH	Parathyroid hormone
PTE	Parathyroid extract
PHPT	Primary hyperparathyroidism
SHPT	Secondary hyperparathyroidism
CDN	Cortical distal nephron
[Ca]_s_	Serum calcium concentration, mass/volume
[Ca]_i_	Serum ionized calcium concentration, mass/volume
[Ca]_uf_	Serum ultrafilterable calcium concentration, mass/volume
[Ca]_u_	Urine calcium concentration, mass/volume
[Ca]_CDN_	Calcium concentration in the cortical distal nephron, mass/volume
I_Ca_	Influx of calcium (into extracellular fluid or plasma), mass/time
E_Ca_	Urinary excretion rate of calcium, mass/time
TR_Ca_	Rate of tubular reabsorption of calcium, mass/time
E_Ca_/C_cr_	Amount of calcium excreted per volume of filtrate, mass/volume
TR_Ca_/C_cr_	Amount of calcium reabsorbed per volume of filtrate, mass/volume
[P]_s_	Serum phosphorus concentration, mass/volume
[P]_p_	Plasma phosphorus concentration, mass/volume
[P]_u_	Urine phosphorus concentration, mass/volume
[P]_CDN_	Phosphorus concentration in the cortical distal nephron, mass/volume
I_P_	Influx of phosphorus into extracellular fluid or plasma, mass/time
E_P_	Urinary excretion rate of phosphorus, mass/time
TR_P_	Tubular reabsorption rate of phosphorus, mass/time
E_P_/C_cr_	Amount of phosphorus excreted per volume of filtrate, mass/volume
TR_P_/C_cr_	Amount of phosphorus reabsorbed per volume of filtrate, mass/volume
25D	25-hydroxyvitamin D
1,25D	1,25-dihydroxyvitamin D
EDTA	Ethylenediaminetetraacetic acid
VDRA	Vitamin D receptor activator
mRNA	Messenger RNA
FGF23	Fibroblast growth factor 23
NHE3	Sodium-hydrogen exchanger 3
